# Preimplantation genetic testing for *KIT*-associated pigmentary monogenic diseases

**DOI:** 10.3389/fmed.2026.1947434

**Published:** 2026-10-01

**Authors:** Zhou Zhou, Shanshan Liang, Ningxin Qin, Jianzhi Yang, Mengmeng Tian, Xiaoming Teng, Junyu Zhang

**Affiliations:** 1 Reproductive Medicine Center, Shanghai First Maternity and Infant Hospital, School of Medicine, Tongji University, Shanghai, China; 2 Shanghai Key Laboratory of Maternal Fetal Medicine, Shanghai Institute of Maternal Fetal Medicine and Gynecologic Oncology, Shanghai First Maternity and Infant Hospital, School of Medicine, Tongji University, Shanghai, China

**Keywords:** hyperpigmentation, *KIT*, lentigines, NIPT, PGT, piebaldism

## Abstract

This study addresses the genetic diagnosis and reproductive intervention for families affected by *KIT*-related pigmentary disorders, which can manifest as either piebaldism due to loss-of-function variants or hyperpigmentation due to gain-of-function variants. We recruited three Chinese families presenting with these distinct phenotypes to identify pathogenic variants and prevent intergenerational transmission. Whole exome sequencing and Sanger sequencing identified three heterozygous *KIT* variants: a known missense variant (c.2485G>C) in family 1 causing hyperpigmentation and generalized lentigines, and two novel null variants (c.2736_2737insT and c.2664del) in families 2 and 3 causing piebaldism. To assist these families, we implemented a comprehensive preimplantation genetic testing (PGT) for monogenic diseases and aneuploidies. This approach successfully identified euploid embryos free of pathogenic variants for transfer. Uniquely, in family 1, the PGT results were verified during pregnancy using non-invasive prenatal testing for monogenic diseases (NIPT-M) via maternal plasma cell-free DNA analysis, providing a non-invasive confirmatory screen and potentially reducing the need for invasive procedures. Families 1 and 2 successfully delivered healthy infants. This study demonstrates the first successful application of PGT combined with NIPT-M for preventing *KIT* variant-related pigmentation disorder and enriches the *KIT* gene's mutational spectrum. This integrated approach provides an effective strategy for preventing the transmission of these monogenic conditions and holds promise for wider application.

## Introduction

*KIT* is a proto-oncogene encoding a type III receptor tyrosine kinase for stem cell factors (1). It is predominantly expressed in mast cells, hematopoietic stem cells, germ cells, melanocytes, and the interstitial cells of Cajal (2). The cellular response to KIT signaling is cell type specific (3). KIT is functionally relevant for mast cell development, hematopoiesis, gametogenesis and melanogenesis (2). Particularly in melanocytes, KIT signaling triggers the Ras/MAPK signaling pathway and plays essential roles in their migration, proliferation, differentiation, survival, melanogenesis, and melanosome transfer (4).

Germline variants in the *KIT* gene manifest distinct phenotypes depending on their functional impact. Loss-of-function variants cause piebaldism, an autosomal dominant pigmentation disorder characterized by a congenital white forelock and leukoderma on the frontal scalp, forehead, ventral trunk and extremities (4). These depigmented patches result from improper melanoblast migration during embryo development. Conversely, gain-of-function variants in *KIT* gene induce mastocytosis and gastrointestinal stromal tumors (GISTs) (5). Several cases of hyperpigmentation and lentigines were reported in familial mastocytosis or GISTs associated with germline *KIT* variants (6).

Preimplantation genetic testing for monogenic diseases (PGT-M) offers couples with monogenic diseases the opportunity to prevent disease transmission by analyzing embryos before implantation (7). However, the procedure faces technical challenges due to limited number of cells available from trophectoderm biopsies, raising concerns about allele dropout (ADO) and DNA contamination (8, 9). To enhance diagnostic accuracy, current PGT-M protocols employ next-generation sequencing (NGS) for SNP-based haplotype analysis of regions flanking pathogenic variants (10). This approach requires DNA samples from both couples and the proband for comprehensive linkage analysis. Additionally, embryo aneuploidy rates are widely reported to increase with increasing female age and to cause a lower pregnancy and delivery rate (11, 12). Therefore, PGT for aneuploidies (PGT-A) is often recommended alongside PGT-M to screen for chromosomal abnormalities (13). The combined testing ensures that neither pathogenic variant carriers nor aneuploid embryos are selected for transfer.

The discovery of fetal cell-free DNA (cfDNA) in the maternal plasma has enabled the development of noninvasive prenatal testing for both aneuploidies (NIPT) and monogenic diseases (NIPT-M) (14). Through cfDNA haplotyping analysis, the fetal genome-wide haplotype and copy number can be determined. While detecting paternally inherited alleles is straightforward, analysis of maternally inherited fetal alleles remains challenging due to excess maternal DNA in cfDNA (15).

In this study, we identified a heterozygous variant c.2485G>C in *KIT* from an affected individual clinically diagnosed with hyperpigmentation and lentigines. We also investigated two unrelated families with piebaldism and identified two novel heterozygous *KIT* variants. To prevent intergenerational transmission of these pathogenic variants, we implemented a combined-PGT protocol including PGT-A and PGT-M. We reported the successful application of this comprehensive PGT approach, coupled with NIPT verification, in helping these families achieve healthy pregnancies.

## Methods

### Patients and ethical statement

Three Chinese families with either hyperpigmentation and lentigines or piebaldism were recruited in this study. Peripheral blood samples were collected for genetic analysis. Informed consent was obtained from all patients and their related families before genetic testing. The study was approved by the Ethics Committee of the Hospital.

### Whole exome sequencing and data analysis

The DNA library was constructed by hybridization capture from the genomic DNA extracted from the peripheral blood samples after fragmentation, adapter ligation, ampliﬁcation, and puriﬁcation. The DNA was detected by a high-throughput sequencing platform (mean sequencing depth, 130.19×). The original sequencing data were compared with the UCSC hg19 human reference genome sequence and further ﬁltered out with synonymous variants or small non-frame shift InDel variants in repeat regions. The remaining data were analyzed according to the inheritance patterns and clinical phenotypes. Candidate variations were further veriﬁed by Sanger sequencing. The pathogenicity of genetic variation was classiﬁed according to the ACMG/AMP guidelines (16).

### Ovarian stimulation, ICSI, and blastomere biopsy

The ovarian stimulation, ICSI, embryo culture, and transfer were conducted according to the routine protocols (17–19). The blastocysts were subjected to TE biopsy on day 5 via laser. Approximately 5–10 cells were extracted from each blastocyst and ready for whole genome amplification (WGA). The ICSI, embryo biopsy, WGA, and NGS-based PGT were performed at the Reproductive Center of Shanghai First Maternity and Infant Hospital.

### PGT and embryo transfer

WGA was performed on the biopsied TE cells using the REPLI-g Single Cell Kit (QIAGEN). The WGA products of each embryo were subjected to Sanger sequencing to identify variant directly. During the pretesting, SNPs distributed upstream and downstream within 2 Mb of the variant sites in *KIT* were chosen to determine the embryo genotypes via NGS using DNA samples from the couples and their parents. Besides the detection of *KIT* variant, CNV analysis was also carried out using WGA products. Any deletions or duplications more than 4 Mb and mosaicism more than 30% in each embryo were reported. The selection for embryo transfer was based on the morphology and the PGT results. Clinical pregnancy was defined as the presence of a fetal heartbeat by sonography 35 days after frozen embryo transfer (FET).

### NIPT-M and data analysis

To verify the PGT diagnosis, potential fetal cells were isolated from blood samples of the pregnant women at 13 weeks of gestation. Cell-free DNA (cfDNA) was extracted from maternal plasma using a cfDNA Extraction Kit (DK607, LifeFeng, China). Extracted gDNA and cfDNA was used to construct sequencing library through the Hieff NGS® OnePot DNA Library Prep Kit (12203ES96, Yeasen, China) and VAHTS Universal DNA Library Prep Kit for MGI with VAHTS Dual UMI Adapters.

The NIPT-M target capture panel (Twist bioscience) contains common SNPs localized in the 2 Mb upstream and downstream sequence regions of the *KIT* gene (GRCh38/hg38). 128 SNPs with a minor allele frequency threshold greater than 30% were selected from the Chinese population, with a median inter-locus distance of 17.5 Kb. All hybridization probes were manufactured by Twist Bioscience and targeted enrichment was performed following manufacturer's protocol. The post-capture library was sequenced in 150 bp paired-end (PE) mode on the DNBSEQ-T7 sequencing platform (MGI-tech, China). Raw data were aligned to the human reference genome (GRCh38/hg38) using Sentieon software. Following processing of sort, de-duplicate and variant calling were also performed using Sentieon. After base calling, mean depth of mapped reads on target region were around 75X and 162.5X for gDNA and cfDNA samples, respectively. SNPs that were homozygous for different alleles in both parents and heterozygous in the fetus were selected to estimate the fetal fraction in maternal plasma. NIPT-M is performed through a haplotype-based strategy using Sanger sequencing confirmed variant-carrying embryo as index sample to construct paternal haplotype. Hap0 was defined as the pathogenic haplotype that passed from the affected parent to the fetus, and Hap1 was defined as the normal haplotype. Subsequently, an HMM model was constructed to determine the transmission status of parental haplotypes as published (20).

cfDNA library was also sequenced on DNBSEQ-T7 platform in 150 bp PE mode for NIPT analysis. Sequencing depth was approximately 1x, and the data volume was around 20 million reads. GC correction and PCA normalization was performed, and Z-score testing approach was used to screen for fetal autosomal aneuploidy of trisomy 13, 18 and 21. Fetal CNVs were classified using Stouffer's Z-score method. Each chromosome or microdeletions/microduplications were marked as having chromosome aberrations if they have an absolute Z-score over 3.

## Results

### Clinical characteristics of three Chinese families with pigmentary disorder

We studied three Chinese families presenting with distinct pigmentation disorders. Clinical information of patients was summarized in Supplementary Table 1.

In family 1, the proband exhibited progressive hyperpigmentation and generalized lentigines (Figure 1A). Physical examination revealed hyperpigmentation with a dark brown spot and lentiginous-like lesions distributed across the face, neck, chest, back, inguinal region, hands, and feet with any other systemic manifestations (Figure 1A). His father displayed similar but mild skin symptoms and was diagnosed with GISTs at the age of 54, whereas the proband (aged 30) has not shown any gastrointestinal manifestations to date.

**Figure 1:**
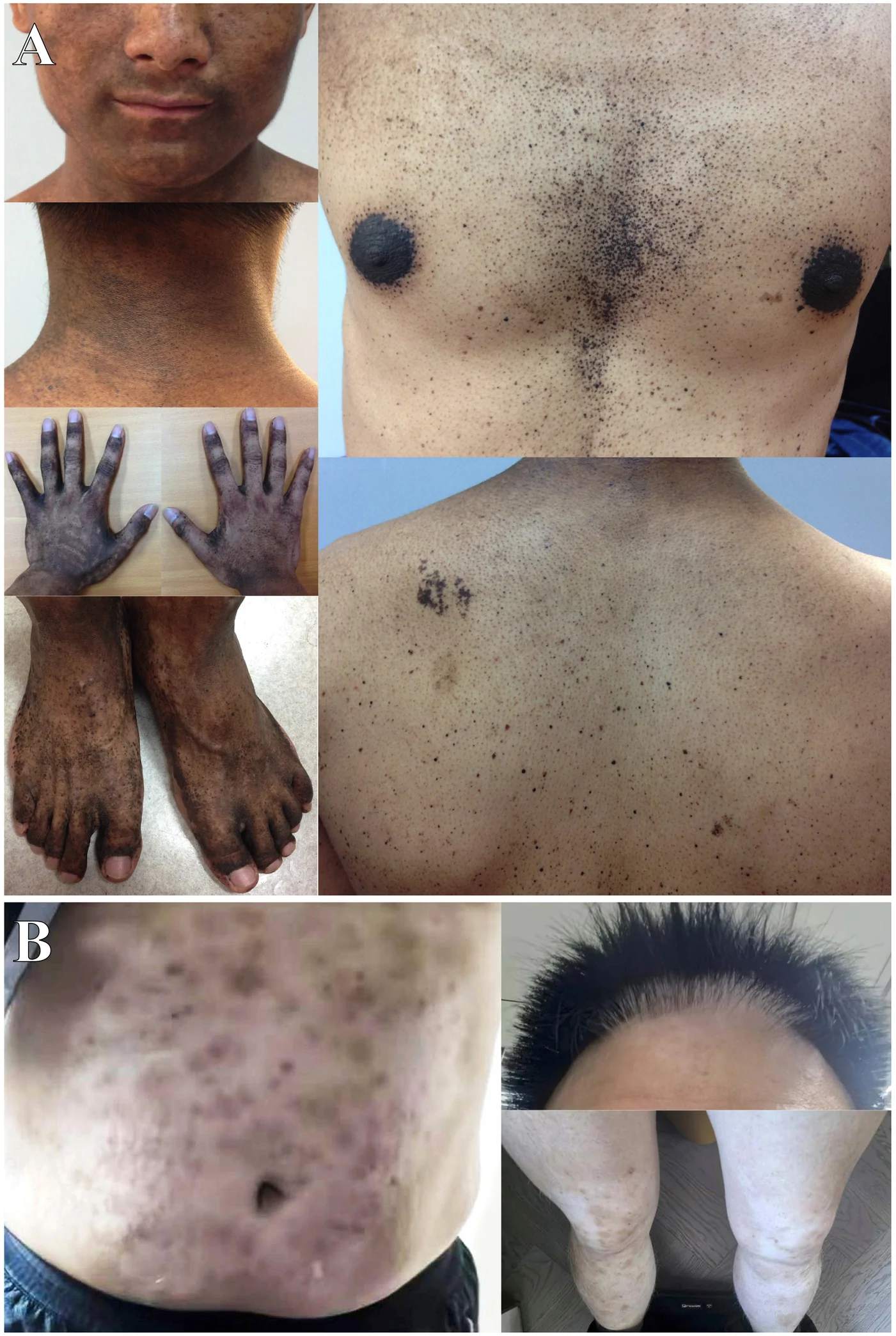
Clinical features of the probands. **(A)** Clinical features of the proband in family 1 showing hyperpigmentation and multiple lentigines on face, neck, chest, back, hands, feet, and inguinal areas. **(B)** The proband in family 2 presented with classic piebaldism features: a congenital white forelock and irregular white patches on the forehead, abdomen, arms, and legs.

Family 2 presented with classic piebaldism: the proband had a congenital white forelock and irregular white patches on the forehead, abdomen, and extremities (Figure 1B), a phenotype also seen in his mother, maternal grandmother, uncle, and cousin.

In family 3, the female proband exhibited white hair near the front hairline and depigmented patches on the forehead and right lower leg, and her father had similar white patches on the forehead, scalp, ventral trunk, and extremities.

### Identification of *KIT* variants in the patients

Through whole exome sequencing, we identified three different heterozygous *KIT* variants. In family 1, we detected a heterozygous missense variant, c.2485G>C (p.Ala829Pro), which was inherited from the proband's father (Figure 2A). The variant was classified as Pathogenic based on ACMG/AMP guidelines and ClinGen recommendations based on multiple lines of evidence: previously reported in the literature (PS4_Supporting), assumed *de novo* in the reported patient (PM6) (21), phenotype and family history were consistent with *KIT*-induced hyperpigmentation and lentigines (PP4) (22), segregated with the phenotype in ﬁve affected patients and was absent in one unaffected individual (PP1_Strong) (22), absence in gnomAD (PM2_Supporting) and *in silico* predictions of pathogenicity (PP3_Moderate).

**Figure 2:**
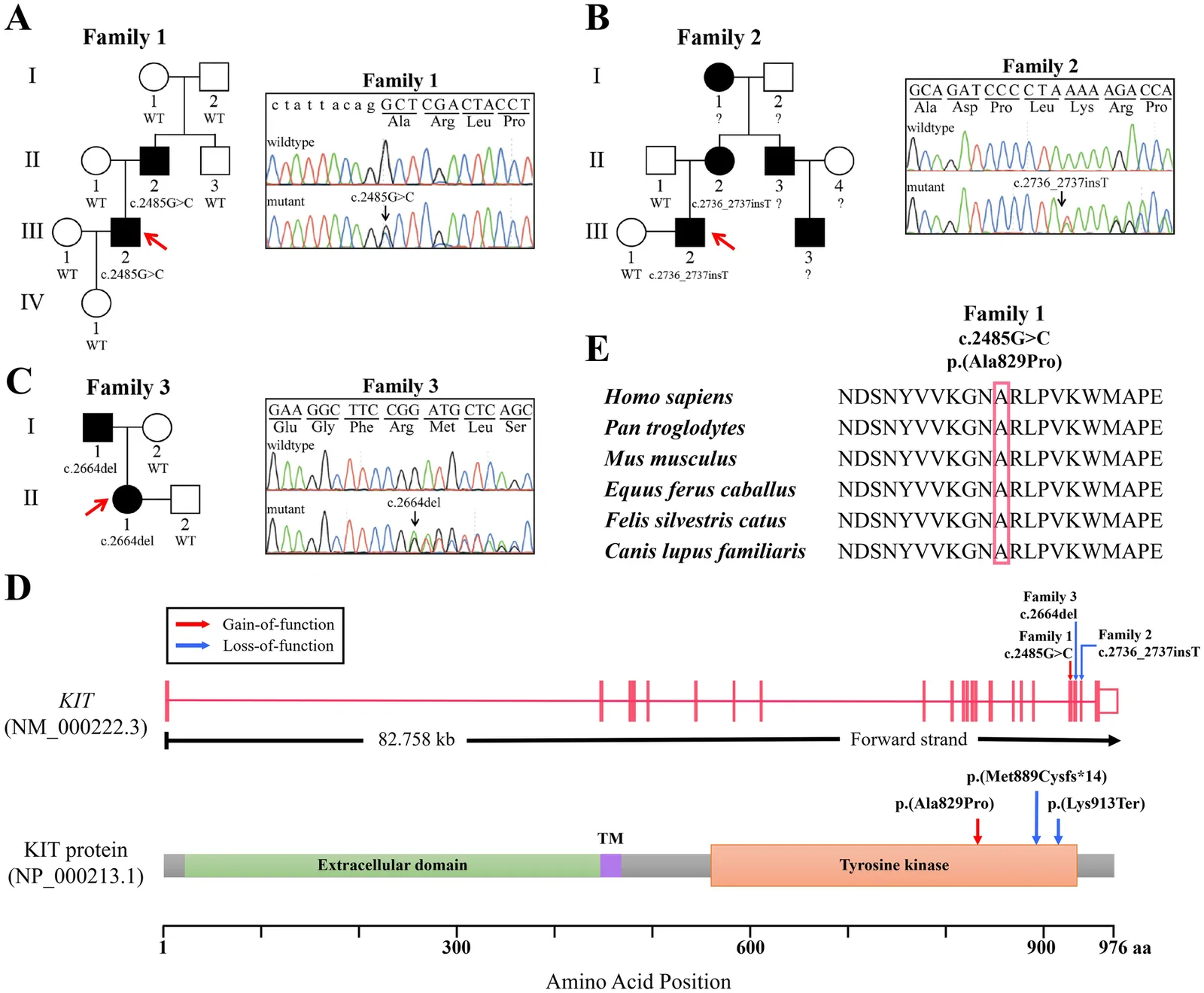
Analysis of *KIT* variants: pedigrees, validation, and conservation. Pedigrees of family 1 **(A)**, family 2 **(B)**, and family 3 **(C)** Three *KIT* variants identiﬁed in 3 families were validated using Sanger sequencing. Nucleotide sequence and corresponding amino acid sequence were presented. Squares and circles indicate males and females, respectively; solid symbols indicate affected patients; open symbols indicate unaffected subjects; red arrows indicate the probands in each family; WT: wild type. **(D)** Location of the *KIT* variants in the gene and protein structure. **(E)** Orthologous protein sequence alignment of KIT sequence. Conservation analysis among six species showed the *KIT* variant c.2485G>C happened in a highly conserved region of KIT among different species.

In families 2 and 3, we identified two novel null variants: a nonsense variant, c.2736_2737insT (p.Lys913Ter), and a frameshift variant, c.2664del (p.Met889Cysfs*14), respectively. Both novel variants were classified as Likely pathogenic based on their predicted impact on protein function (PVS1) and absence from population databases (PM2_Supporting). Sanger sequencing confirmed the segregation of these *KIT* variants with the disease phenotype in all three families (Figures 2B,C).

KIT is composed of an extracellular ligand-binding domain, a transmembrane domain, a juxta-membrane domain, and an intracellular kinase domain (Figure 2D). Orthologous protein sequence alignment showed the missense *KIT* variant c.2485G>C happened in a highly conserved region of KIT among different species (Figure 2E). Additionally, a dedicated gain-of-function prediction using the GoFCards database yielded a “GOF predicted” score of 0.88 (scale 0–1, higher = more likely GOF), further supporting a gain-of-function mechanism for p.Ala829Pro (23). The location and basic information of all variants are summarized in Table 1.

**Table 1:** Classiﬁcation of pathogenicity of *KIT* variants identiﬁed from three families.

Family	Genomic position on chr4 (bp)	*KIT* variants (NM_000222.3)	ACMG classiﬁcation	Criteria applied	Inheritance and zygosity	Allele frequency in gnomAD	Revel	Mutation Taster	Reported before or not
1	55,602,664	c.2485G>C p.(Ala829Pro)	Pathogenic	PS4_Supporting; PM6; PP4; PP1_Strong; PM2_Supporting; PP3_Moderate	Heterozygote, paternally inherited	0	Damaging (0.787)	Disease_causing (1)	Lu Yang et al., 2022 (1)
2	55,603,380	c.2736_2737insT p.(Lys913Ter)	Likely pathogenic	PVS1; PM2_Supporting	Heterozygote, maternally inherited	0	—	Disease_causing (1)	This study
3	55,602,952	c.2664del p.(Met889Cysfs*14)	Likely pathogenic	PVS1; PM2_Supporting	Heterozygote, paternally inherited	0	—	Disease_causing (1)	This study

### PGT in three Chinese families with *KIT* variants

Following genetic counseling, all couples opted for a comprehensive protocol combining PGT-M and PGT-A. The PGT cycles yielded successful outcomes (Table 2). In family 1, ICSI yielded eight normally fertilized oocytes from 19 retrieved, resulting in five blastocysts. All of them were biopsied; PGT-A revealed three were euploid, and PGT-M confirmed that two of these (E1–1 and E1–3) were free of the familial *KIT* variant (Supplementary Figure 1 and Table 2). Based on comprehensive evaluation, embryo E1–3 was transferred, leading to a successful pregnancy. For family 2, nine blastocysts were tested, leading to the identification of unaffected, euploid embryo E3–1 (Supplementary Figure 2 and Table 3), which was transferred and ultimately resulted in the delivery of a healthy infant at 39 weeks gestation. In family 3, sixteen blastocysts underwent testing, and an unaffected euploid embryo (E2-3) was transferred (Supplementary Figure 3 and Table 4). Although this pregnancy was terminated at 23 weeks due to an unrelated diagnosis of amniotic band syndrome, subsequent analysis confirmed the fetus was chromosomally normal and did not carry the pathogenic *KIT* variant, validating the PGT result.

**Table 2:** Summary of PGT results for embryos.

Case ID	*KIT* variants	Cycle	Oocyte retrieved	Fertilized oocytes	Biopsied Embryos	Embryo ID	PGT-A results	PGT-M results		Clinical outcomes
								Sanger sequencing	Haplotype analysis	
1	c.2485G>C	1	19	8	5	E1-1	Euploid	WT	WT	Frozen
						E1-2	Euploid	Carrier	Carrier	Abandoned
						E1-3^a^	Euploid	WT	WT	Live birth
						E1-4/5	Aneuploid	WT	WT	Abandoned
2	c.2736_2737insT	1	28	19	5	E1-1	Aneuploid	Carrier	Carrier	Abandoned
						E1-2/3/5	Aneuploid	WT	WT	Abandoned
						E1-4	Euploid	Carrier	Carrier	Abandoned
		2	18	8	2	E2-1	Aneuploid	Carrier	Carrier	Abandoned
						E2-2	Aneuploid	WT	WT	Abandoned
		3	14	9	2	E3-1^a^	Euploid	WT	WT	Live birth
						E3-2	Euploid	Carrier	Carrier	Abandoned
3	c.2664del	1	21	15	9	E1-1/2/3/6/7/9	Euploid	Carrier	Carrier	Abandoned
						E1-4/5	Aneuploid	Carrier	Carrier	Abandoned
						E1-8	Aneuploid	WT	WT	Abandoned
		2	17	14	7	E2-1/6	Euploid	WT	WT	Frozen
						E2-2	Aneuploid	Carrier	Carrier/WT^b^	Abandoned
						E2-3^a^	Euploid	WT	WT	Terminated pregnancy
						E2-4	Aneuploid	Carrier	Carrier	Abandoned
						E2-5	Euploid	Carrier	Carrier	Abandoned
						E2-7	Aneuploid	WT	WT	Abandoned

WT, wild type.

Haplotype analysis: During the pretesting, SNPs distributed upstream and downstream within 2 Mb of the variant sites in *KIT* were chosen to determine the embryo genotypes via NGS using DNA samples from the couples and their parents.

a The euploid embryo without heterozygous variant was transferred into uterus.

b Haplotype analysis was performed to define the haplotype blocks derived from paternal homolog 1 (H1) and H2 or maternal H1 and H2, thus indicating homologous recombination sites between the parental H1 and H2.

### Prenatal diagnosis and successful pregnancy outcome

A key innovation of our approach was the use of NIPT-M to confirm the PGT-M results in family 1 (20). Patients undergoing PGT are often reluctant to pursue invasive prenatal diagnostics due to associated risks. NIPT-M offers a non-invasive confirmatory screening option, although it is not a diagnostic equivalent to invasive prenatal diagnosis. In family 1, at 13-week of gestation, cell-free DNA (cfDNA) was extracted from maternal plasma, with a fetal fraction of 14.5%. The sequencing depth for the targeted region is 199.8× at average, ranging from 133× to 286×. And the analytical sensitivity for detecting the paternal wild-type haplotype is 98.7%. A haplotype-based strategy confirmed that the fetus inherited the father's normal haplotype and did not carry the pathogenic c.2485G>C variant. Furthermore, the NIPT results also showed a low risk for trisomies 21, 18, and 13, and no pathogenic or likely pathogenic copy number aberrations were found within the scope of the test. These reassuring results were followed by the birth of a healthy female infant at 38 weeks. Regarding the absence of NIPT-M in families 2 and 3, the couples had full confidence in the PGT results and, considering the additional economic cost, voluntarily declined further non-invasive prenatal confirmation.

## Discussion

In this study, we have detailed the successful application of a comprehensive reproductive strategy, integrating PGT-M, PGT-A, and NIPT-M, to prevent the transmission of *KIT*-related pigmentation disorders. To our knowledge, this represents the first report of successful PGT combined with NIPT-M specifically for these conditions.

The *KIT* gene plays a critical role in melanogenesis, and germline variants can lead to distinct phenotypes such as piebaldism from loss-of-function variants or hyperpigmentation, sometimes associated with GISTs, from gain-of-function variants. Our work contributes to the understanding of KIT pathology by identifying three heterozygous variants in the affected families, including two novel variants (c.2736_2737insT and c.2664del). This expands the mutational spectrum of the *KIT* gene and aids in molecular diagnosis and genetic counseling for these pigmentary disorders. Notably, while the proband in family 1 (aged 30 years) currently exhibits no clinical signs of GIST, his father (aged 54 years) was diagnosed with GISTs. Given that *KIT*-related GISTs typically manifest between 40 and 60 years of age (24), long-term clinical surveillance, including regular gastrointestinal evaluations, is strongly recommended for this proband to enable early detection and intervention should GISTs develop in the future.

PGT-M offers a valuable option for families seeking to avoid the transmission of known monogenic diseases by allowing for the selection of unaffected embryos prior to implantation. We addressed potential technical challenges of PGT-M, such as allele dropout and DNA contamination, by employing SNP-based haplotype analysis. Furthermore, the concurrent application of PGT-A allowed for the screening of aneuploidies, which is a significant factor in achieving successful pregnancies.

A key aspect of our approach was non-invasive confirmatory screen using NIPT-M. Pregnant women who have undergone PGT are often concerned about the risk of miscarriage associated with invasive prenatal diagnostic procedures and are therefore typically reluctant to undergo them. In this context, the use of NIPT-M in family 1 to confirm the PGT results provides an exemplary model for such PGT families. NIPT-M accurately verified the embryo's carrier status from maternal plasma cfDNA, providing crucial reassurance and confirming the healthy status of the fetus regarding the familial *KIT* variant. It is important to emphasize that NIPT-M is currently an advanced screening modality rather than a diagnostic test equivalent to amniocentesis or chorionic villus sampling. A high-risk NIPT-M result would still require invasive prenatal diagnosis for definitive confirmation. In addition, detection of maternally inherited variants remains technically challenging due to the excess maternal cfDNA background; in this study, the family 1 variant was paternally inherited, which facilitated haplotype-based analysis. For maternally inherited variants, more sophisticated analytical strategies and cautious interpretation are required.

In conclusion, this study demonstrates the first successful application of PGT combined with NIPT-M for preventing *KIT* variant-related pigmentation disorder. By combining SNP-based PGT-M for accuracy, PGT-A for euploidy selection, and NIPT-M for safe, non-invasive confirmation, our approach addresses key clinical and technical challenges in assisted reproduction for monogenic diseases. This work not only enriches the mutational spectrum of the *KIT* gene but also provides a promising and broadly applicable strategy for other families affected by pigmentary monogenic disorders.

## Data Availability

The datasets presented in this study can be found in online repositories. The names of the repository/repositories and accession number(s) can be found in the article/Supplementary Material.
