# Supplementary material for: Preimplantation genetic testing for KIT-associated pigmentary monogenic diseases
Source: Front Med (Lausanne). 2026 Oct 1;13:1947434. doi: 10.3389/fmed.2026.1947434 (PMC13668847; doi:10.3389/fmed.2026.1947434)
Supplement: Supplementary file 1 [file Supplementaryfile1.docx]

**Supplemental Figure 1** Copy number variations **(A)** and Sanger sequencing results **(B)** of the embryos from case 1

**Supplemental Figure 2** Copy number variations **(A)** and Sanger sequencing results **(B)** of the embryos from case 2

**Supplemental Figure 3** Copy number variations **(A)** and Sanger sequencing results **(B)** of the embryos from case 3

**Supplemental Table 1.** Clinical characteristics of the affected individuals in three families

| **Family** | **Individual No.** | **Sex** | **Age (years)** | **Hyperpigmentation and lentigines** | **Gastrointestinal stromal tumors (GISTs)** | **Piebaldism** |
| --- | --- | --- | --- | --- | --- | --- |
| 1 | III-2 | Male | 30 | Heavy | NA | NA |
|  | II-2 | Male | 54 | Mild | Yes | NA |
| 2 | III-2 | Male | 28 | NA | NA | Yes |
|  | II-2 | Female | 51 | NA | NA | Yes |
| 3 | II-1 | Female | 29 | NA | NA | Yes |

**Supplemental Table 2.** Representative SNPs haplotyping results for family 1

| **Chromosome number** | **Genomic position** | **Reference SNP cluster ID*** | **III-1** | **III-2^#^** | **II-2** | **E1-1** | **E1-2** | **E1-3** | **E1-4** | **E1-5** |
| --- | --- | --- | --- | --- | --- | --- | --- | --- | --- | --- |
| chr4 | 53724744 | rs6818178 | G/G | A/G | G/G | G/A | G/G | G/A | G/A | G/A |
| chr4 | 54330867 | rs2412488 | G/G | A/G | A/G | G/A | G/G | G/A | G/A | G/A |
| chr4 | 54603082 | rs79422632 | A/A | A/C | A/C | A/A | A/C | A/A | A/A | A/A |
| chr4 | 55390494 | rs170118 | C/C | C/A | C/A | C/C | C/A | C/C | C/C | C/C |
| chr4 | 55599436 | rs1008658 | C/C | C/T | C/T | C/C | C/T | C/C | C/C | C/C |
| *KIT* (NM_000222.3, chr4:55602664) | | | | | | | | | | |
| chr4 | 55613382 | rs7676198 | C/C | C/T | C/T | C/C | C/T | C/C | C/C | C/C |
| chr4 | 55986238 | rs4576072 | T/T | T/C | T/C | T/T | T/C | T/T | T/T | T/T |
| chr4 | 56702299 | rs2899039 | G/G | A/G | G/G | G/A | G/G | G/A | G/A | G/A |
| chr4 | 57157682 | rs6833800 | A/A | A/G | A/G | A/A | A/G | A/A | A/A | A/A |
| chr4 | 57606688 | rs4865149 | C/C | C/A | C/A | C/C | C/A | C/C | C/C | C/C |
| *Representative upstream and downstream SNP markers in the adjacent regions of the *KIT* gene were selected to identify the disease-carrying allele in each embryo.  ^#^In the proband of family 1, the pathogenic haplotype is shown in red, and the normal allele is shown in green. | | | | | | | | | | |

**Supplemental Table 3. Representative SNPs haplotyping results for** family **2**

| **Chromosome number** | **Genomic position** | **Reference SNP cluster ID*** | **III-1** | **III-2^#^** | **II-2** | **E1-1** | **E1-2** | **E1-3** | **E1-4** | **E1-5** | **E2-1** | **E2-2** | **E3-1** | **E3-2** |
| --- | --- | --- | --- | --- | --- | --- | --- | --- | --- | --- | --- | --- | --- | --- |
| chr4 | 53710386 | rs7675322 | A/A | A/C | A/C | A/C | A/A | A/A | A/C | A/A | A/C | A/A | A/A | A/C |
| chr4 | 54310508 | rs55849034 | C/C | T/C | C/C | C/C | C/T | C/T | C/C | C/T | C/C | C/T | C/T | C/C |
| chr4 | 54986912 | rs6850083 | T/T | C/T | T/T | T/T | T/C | T/C | T/T | T/C | T/T | T/C | T/C | T/T |
| chr4 | 55139771 | rs35597368 | T/T | T/C | T/C | T/C | T/T | T/T | T/C | T/T | T/C | T/T | T/T | T/C |
| chr4 | 55574182 | rs3020826 | T/T | C/T | T/T | T/T | T/C | T/C | T/T | T/C | T/T | T/C | T/C | T/T |
| *KIT* (NM_000222.3, chr4:55603380-55603381) | | | | | | | | | | | | | | |
| chr4 | 55711761 | rs80267845 | T/T | C/T | T/T | T/T | T/C | T/C | T/T | T/C | T/T | T/C | T/C | T/T |
| chr4 | 56202973 | rs6854287 | G/G | G/A | G/A | G/A | G/G | G/G | G/A | G/G | G/A | G/G | G/G | G/A |
| chr4 | 56663855 | rs2726737 | A/A | A/C | A/C | A/C | A/A | A/A | A/C | A/A | A/C | A/A | A/A | A/C |
| chr4 | 57125816 | rs11944920 | G/G | A/G | G/G | G/G | G/A | G/A | G/G | G/A | G/G | G/A | G/A | G/G |
| chr4 | 57600552 | rs12645925 | T/T | T/C | T/C | T/C | T/T | T/T | T/C | T/T | T/C | T/T | T/T | T/C |
| *Representative upstream and downstream SNP markers in the adjacent regions of the *KIT* gene were selected to identify the disease-carrying allele in each embryo.  ^#^In the proband of family 2, the pathogenic haplotype is shown in red, and the normal allele is shown in green. | | | | | | | | | | | | | | |

**Supplemental Table 4.** Representative SNPs haplotyping results for family 3

| **Chromosome number** | **Genomic position** | **Reference SNP cluster ID*** | **II-1^#^** | **II-2** | **I-1** | **E1-1** | **E1-2** | **E1-3** | **E1-4** | **E1-5** | **E1-6** | **E1-7** | **E1-8** | **E1-9** | **E2-1** | **E2-2** | **E2-3** | **E2-4** | **E2-5** | **E2-6** | **E2-7** |
| --- | --- | --- | --- | --- | --- | --- | --- | --- | --- | --- | --- | --- | --- | --- | --- | --- | --- | --- | --- | --- | --- |
| chr4 | 55036293 | rs7659453 | C/T | T/T | T/T | T/T | T/T | T/T | T/T | T/T | T/T | T/T | T/C | T/T | T/C | T/T | T/C | T/T | T/T | T/C | T/C |
| chr4 | 55143286 | rs1316926 | G/A | A/A | A/A | A/A | A/A | A/A | A/A | A/A | A/A | A/A | A/G | A/A | A/G | A/A | A/G | A/A | A/A | A/G | A/G |
| chr4 | 55478047 | rs2537883 | T/C | C/C | C/C | C/C | C/C | C/C | C/C | C/C | C/C | C/C | C/T | C/C | C/T | C/C | C/T | C/C | C/C | C/T | C/T |
| chr4 | 55569146 | rs3020825 | T/C | C/C | C/C | C/C | C/C | C/C | C/C | C/C | C/C | C/C | C/T | C/C | C/T | C/C | C/T | C/C | C/C | C/T | C/T |
| chr4 | 55582397 | rs17084687 | T/C | C/C | C/C | C/C | C/C | C/C | C/C | C/C | C/C | C/C | C/T | C/C | C/T | C/C | C/T | C/C | C/C | C/T | C/T |
| *KIT* (NM_000222.3, chr4:55602954) | | | | | | | | | | | | | | | | | | | | | |
| chr4 | 55788140 | rs17709654 | A/G | A/A | A/G | A/G | A/G | A/G | A/G | A/G | A/G | A/G | A/A | A/G | A/A | A/A | A/A | A/G | A/G | A/A | A/A |
| chr4 | 55867442 | rs12502309 | C/A | C/C | C/A | C/A | C/A | C/A | C/A | C/A | C/A | C/A | C/C | C/A | C/C | C/C | C/C | C/A | C/A | C/C | C/C |
| chr4 | 56001834 | rs6856677 | T/C | T/T | T/C | T/C | T/C | T/C | T/C | T/C | T/C | T/C | T/T | T/C | T/T | T/T | T/T | T/C | T/C | T/T | T/T |
| chr4 | 56148989 | rs4407562 | C/T | C/C | C/T | C/T | C/T | C/T | C/T | C/T | C/T | C/T | C/C | C/T | C/C | C/C | C/C | C/T | C/T | C/C | C/C |
| chr4 | 56301369 | rs1801260 | A/G | A/A | A/G | A/G | A/G | A/G | A/G | A/G | A/G | A/G | A/A | A/G | A/A | A/A | A/A | A/G | A/G | A/A | A/A |
| *Representative upstream and downstream SNP markers in the adjacent regions of the *KIT* gene were selected to identify the disease-carrying allele in each embryo.  ^#^In the proband of family 3, the pathogenic haplotype is shown in red, and the normal allele is shown in green. | | | | | | | | | | | | | | | | | | | | | |
